# Supplementary material for: Pregnane X receptor activation constrains mucosal NF-κB activity in active inflammatory bowel disease
Source: PLoS One. 2019 Oct 3;14(10):e0221924. doi: 10.1371/journal.pone.0221924 (PMC6776398; doi:10.1371/journal.pone.0221924)
Supplement: S1 Fig — (DOCX) [file pone.0221924.s001.docx]

**
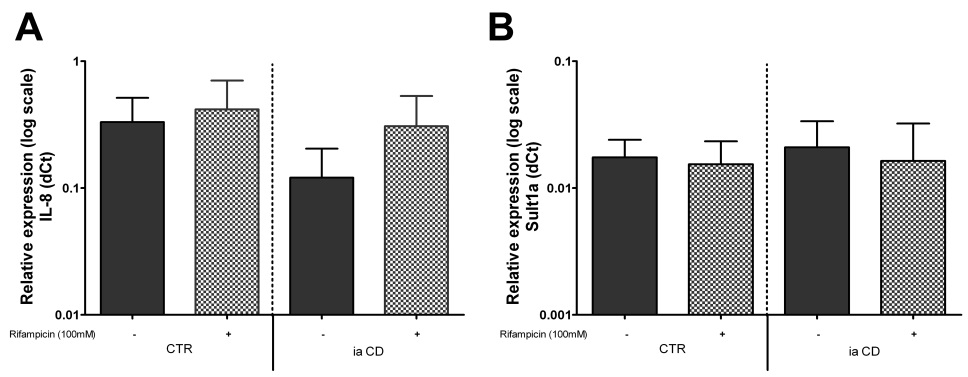
**

**S1 Fig: The effect of rifampicin on PBMC**

A) IL-8 mRNA expression in PBMC. PBMC isolated from peripheral blood samples of CD patients and healthy individuals were stimulated for 18h at 37^o^C with solvent (0.1% (v/v) DMSO) or 100 mM rifampicin. Ctr is the mean IL-8 expression of the healthy individuals (n=4) and ia CD is the mean IL-8 expression of the inactive CD patients (n=5). The error bar is SD.

B) Sult1a mRNA expression in PBMC. Same methodology as used in A was used.
