# Supplementary material for: Pregnane X receptor activation constrains mucosal NF-κB activity in active inflammatory bowel disease
Source: PLoS One. 2019 Oct 3;14(10):e0221924. doi: 10.1371/journal.pone.0221924 (PMC6776398; doi:10.1371/journal.pone.0221924)
Supplement: S2 Fig — (DOCX) [file pone.0221924.s002.docx]

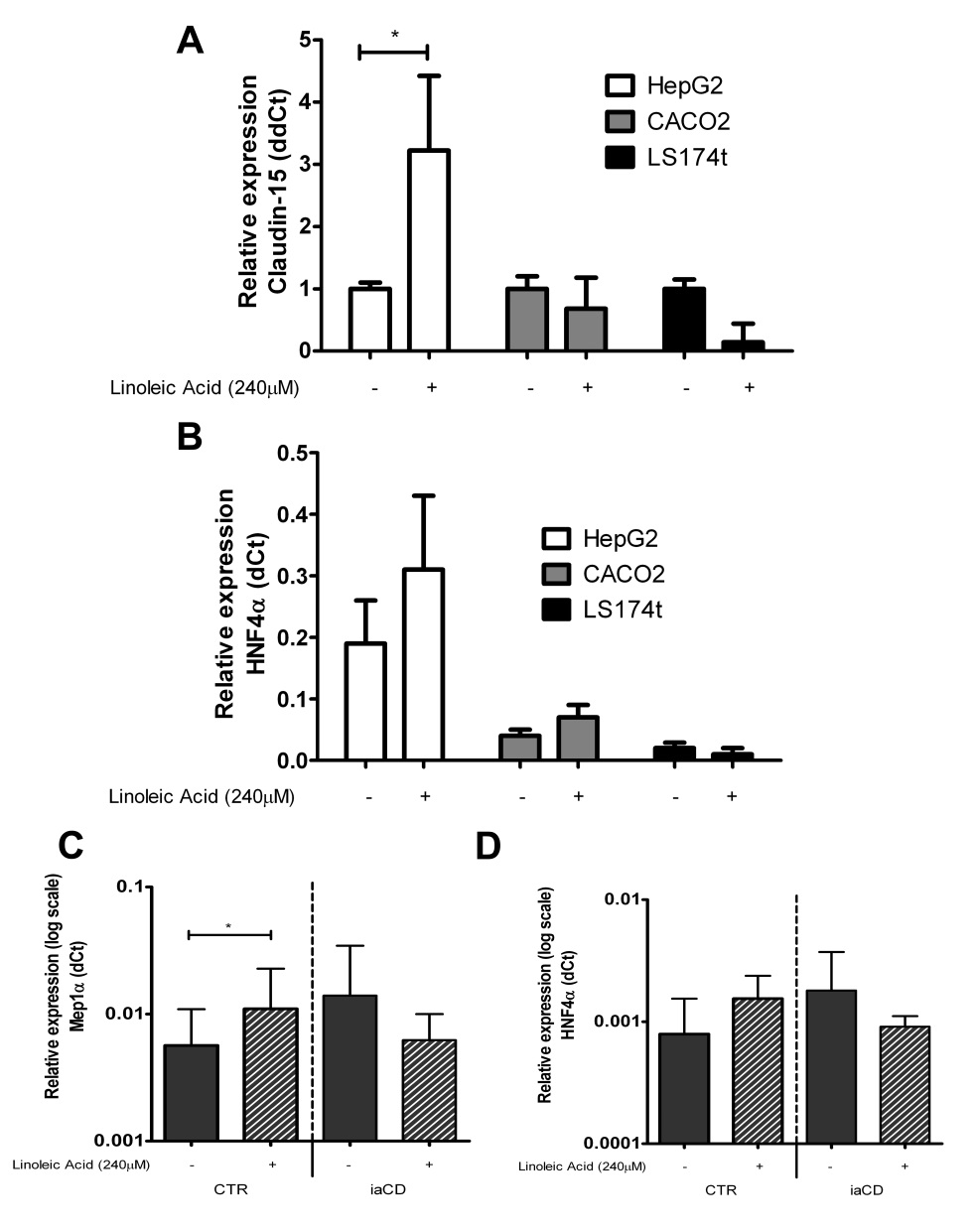


**S2 Fig: Cancer cells with HNF4α stimulation**

A) HNF4α target gene Claudin-15 expression in cancer cell lines. Hepatocyte cancer cell line HepG2 and colon cancer cell line CACO2 and LS174t were stimulated with 240 μM linoleic acid for 18h at 37^o^C. The graph represents the mean relative Claudin-15 expression per cell lines and with or without linoleic acid stimulation line. The error bar is SD, * p<0.05.

B) HNF4α expression in cancer cell lines. Same methodology as in A was used. The graph represents the mean expression of HNF4α per cell line. The error bar is SD.

C) HNF4α target gene Mep1α expression in biopsies. Biopsies from two CD patients and three healthy individuals were stimulated with solvent (1% (v/v) PBS) or 240 μM Linoleic acid for 18 h at 37^o^C. The graph represents the mean Mep1α expression per group. CTR are the controls and iaCD are CD patients with quiescent disease. The error bas is SD.

D) HNF4α expression in biopsies. Same methodology as in C) was used. The graph represents the mean HNF4α expression per group. The error bas is SD.
