# Supplementary material for: Pregnane X receptor activation constrains mucosal NF-κB activity in active inflammatory bowel disease
Source: PLoS One. 2019 Oct 3;14(10):e0221924. doi: 10.1371/journal.pone.0221924 (PMC6776398; doi:10.1371/journal.pone.0221924)
Supplement: S3 Fig — (DOCX) [file pone.0221924.s003.docx]

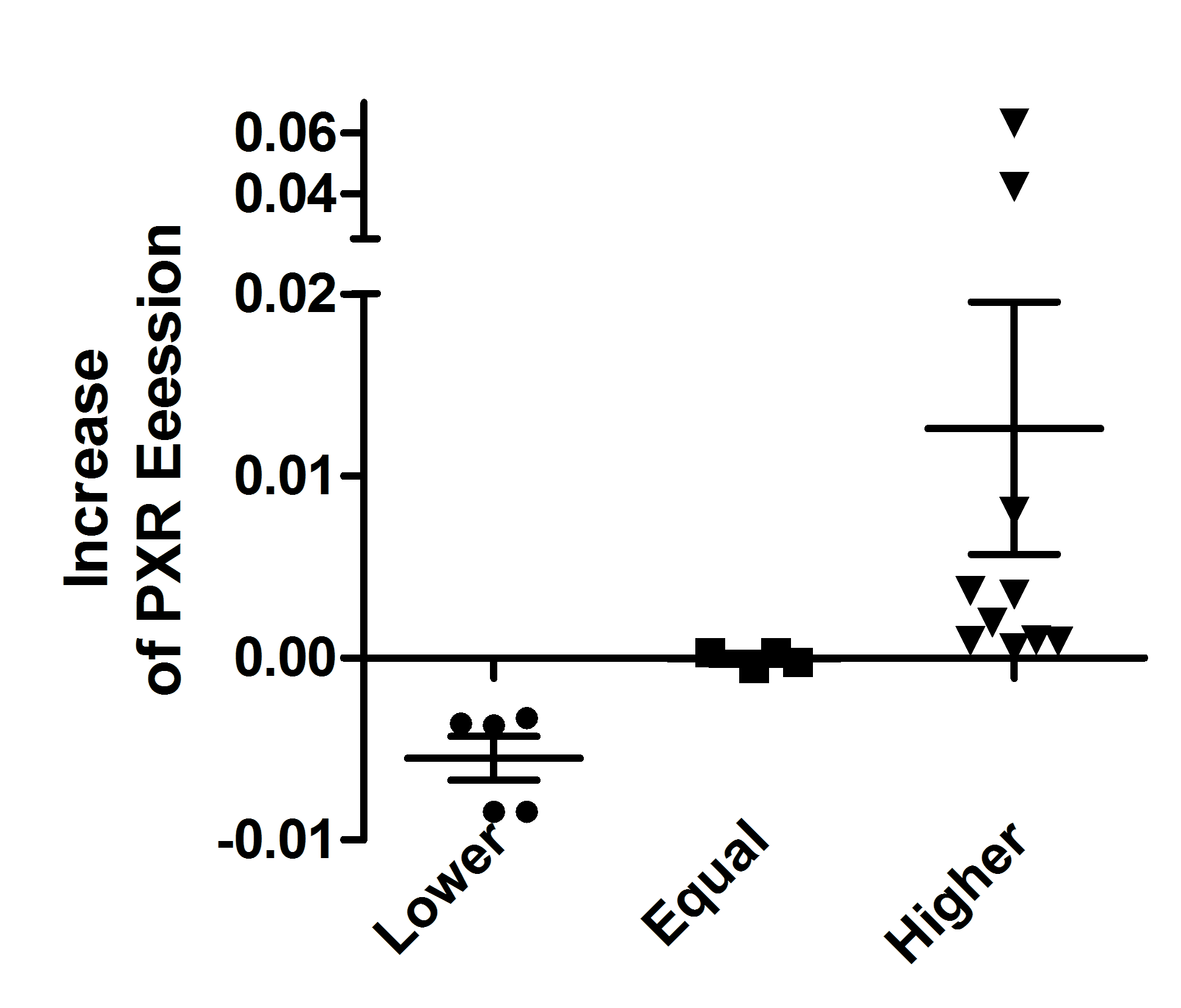


**Figure S3: The increase of PXR mRNA expression by Rifampicin treatment**

19 samples were treated with Rifampicin to induce the expression of PXR. The samples were divided into three groups: Group 1 (n=5) show lower PXR expression after the Rifampicin treatment; Group 2 (n=4) have equal expression of PXR before and after Rifampicin treatment; Group 3 (n=10) have increased PXR expression after the Rifampicin treatment. The graph represents the mean increase of PXR expression. The error bar is SEM.
