# Supplementary material for: Pregnane X receptor activation constrains mucosal NF-κB activity in active inflammatory bowel disease
Source: PLoS One. 2019 Oct 3;14(10):e0221924. doi: 10.1371/journal.pone.0221924 (PMC6776398; doi:10.1371/journal.pone.0221924)
Supplement: S4 Fig — (DOCX) [file pone.0221924.s004.docx]

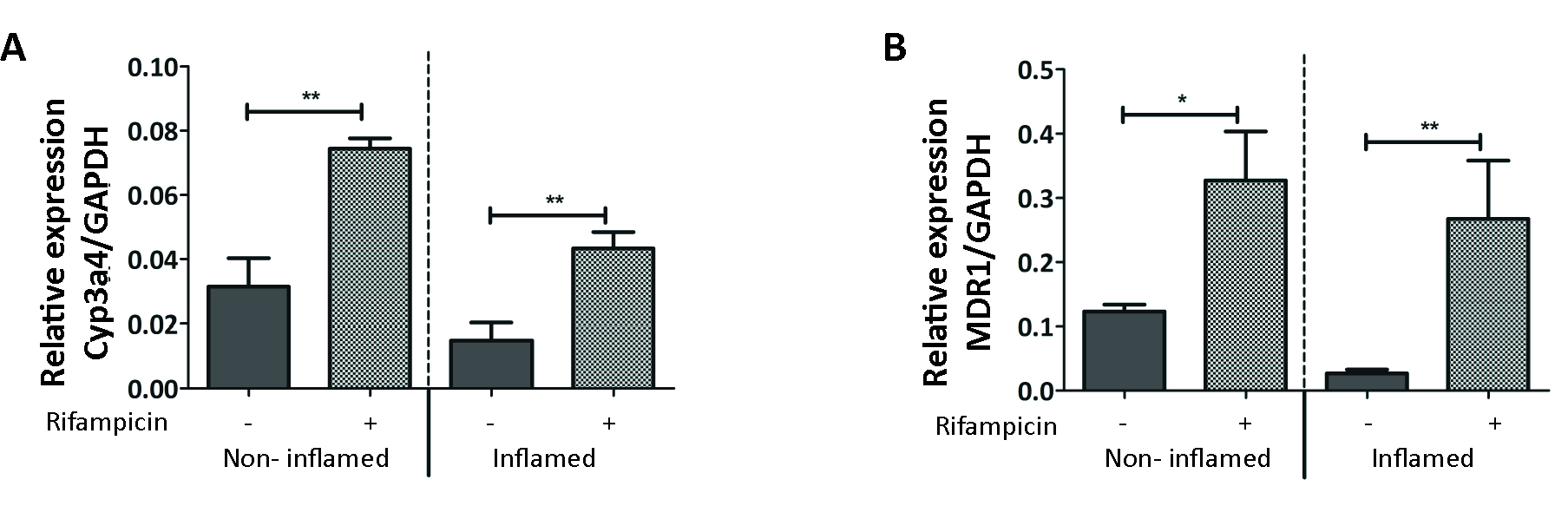


**S4 Fig: The expression levels of PXR target genes in organoids**

A) & B) Cyp3a4 and MDR1 expression in the intestinal organoids from the patient with inflammation bowel disease. The non-inflamed group represents the organoid derived from non-inflamed tissue and the inflamed group represents the organoid derived from inflamed tissue of the same patient. Error bar is SEM. Organoids were cultured with (+) or without (-) Rifampicin. *p < 0.05, ** p<0.01.
