# Supplementary material for: Pregnane X receptor activation constrains mucosal NF-κB activity in active inflammatory bowel disease
Source: PLoS One. 2019 Oct 3;14(10):e0221924. doi: 10.1371/journal.pone.0221924 (PMC6776398; doi:10.1371/journal.pone.0221924)
Supplement: S5 Fig — (DOCX) [file pone.0221924.s005.docx]

**
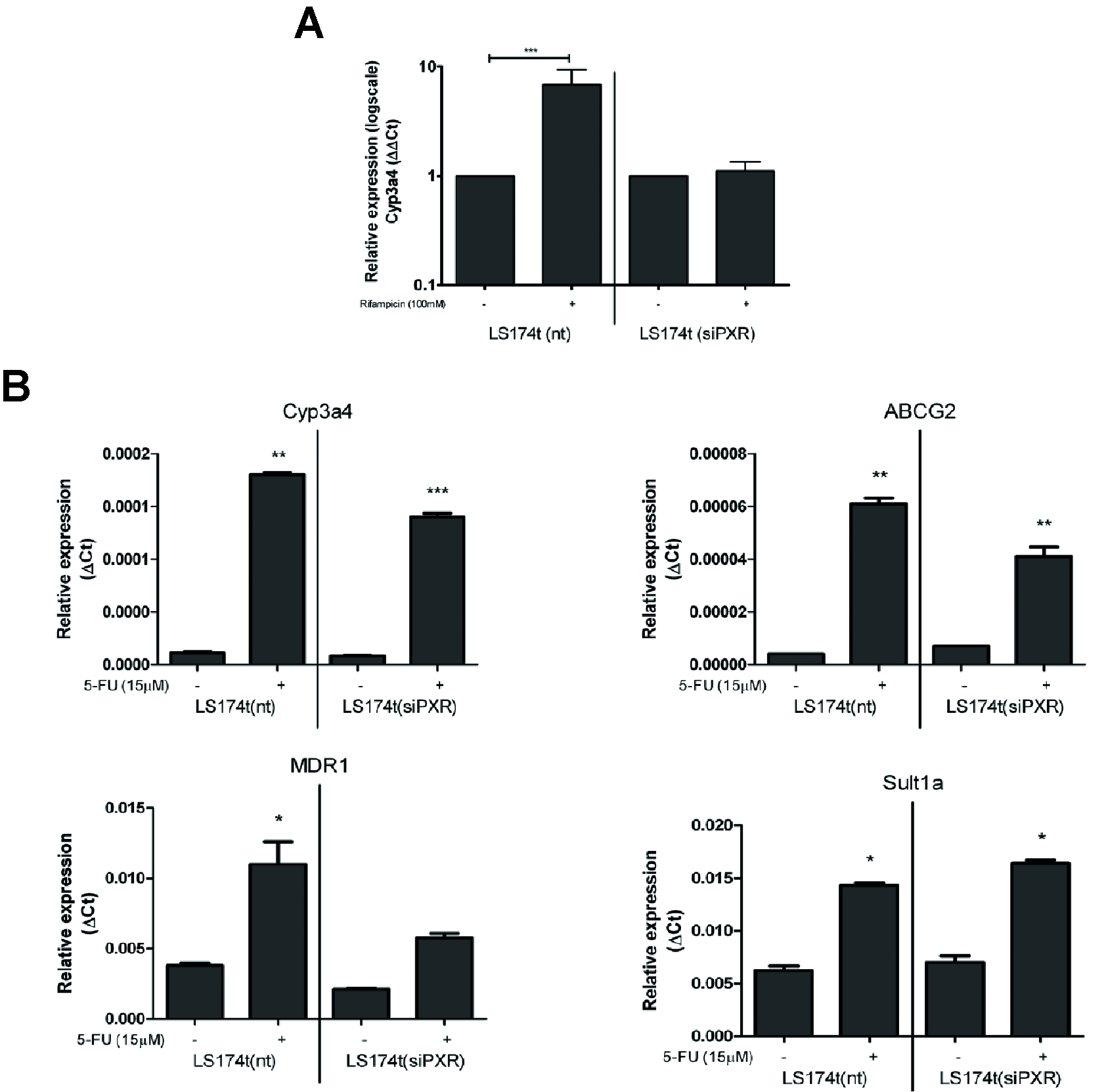
**

**S5 Fig: The expression levels of PXR target genes**

A) PXR specific stimulation by 100 mM rifampicin. This graph represents the average, of two independent experiments performed in duplo, mRNA expression of Cyp3a4. Error bar is SD, *** p<0.001.

B) The expression of multi drug resistant associated genes. The mRNA expression of the indicated genes was measured in the LS174t (nt) cells and the LS174t (siPXR) cells stimulated with 15 mM 5-FU for 72 h. These graphs represent the average of two independent experiments. Error bar is SD. The increase in expression was compared per cell line to the un-stimulated cells using a non-parametric t-test, *p < 0.05, ** p<0.01 and ***p<0.001.
