# Supplementary material for: Pregnane X receptor activation constrains mucosal NF-κB activity in active inflammatory bowel disease
Source: PLoS One. 2019 Oct 3;14(10):e0221924. doi: 10.1371/journal.pone.0221924 (PMC6776398; doi:10.1371/journal.pone.0221924)
Supplement: S1 Table — (DOCX) [file pone.0221924.s006.docx]

| **Table S1: Primer sequences** | |
| --- | --- |
| **Primer name** | **Sequence from 5’ to 3’** |
| GapdH_fw | GCATTGCCCTCAACGACCAC |
| GapdH_rev | CCACCACCCTGTTGCTGTAG |
| YWHAS_fw | ACTTTTGGTACATTGTGGCTTCAA |
| YWHAS_rev | CCGCCAGGACAAACCAGTAT |
| IL8_fw | CACTGCGCCAACACAGAAATTA |
| IL8_rev | ACTTCTCCACAACCCTCTGCAC |
| IL1β_fw | CCCTAAACAGATGAAGTGCTCCTT |
| IL1β _rev | GTAGCTGGATGCCGCCAT |
| Cyp3a4_fw | CAGGAGGAAATTGATGCAGTTTT |
| Cyp3a4_rev | GTCAAGATACTCCATCTGTAGCACAGT |
| HNF4α_fw | ACATGGACATGGCCGACTAC |
| HNF4α _rev | TGCCTCAATCTGGCGAGACG |
| Claudin-15_fw | TGAGGTGGGTGGATTACTTG |
| Claudin-15_rev | TGTTGAAGGCGTACCAGGAG |
| Mep1α_fw | TCAAGCCCTATGAAGGAGAG |
| Mep1α _rev | CCTTATAGGCACATCCTTGG |
| Sult1a_fw | GCACCCACCCTGTTCTCTAC |
| Sult1a_rev | ACCACGAAGTCCACGGTCTC |
| PXR_fw | ATGGCAGTGTCTGGAACTAC |
| PXR_rev | CAGTTGACACAGCTCGAAAG |
|  |  |
