# Supplementary material for: Pregnane X receptor activation constrains mucosal NF-κB activity in active inflammatory bowel disease
Source: PLoS One. 2019 Oct 3;14(10):e0221924. doi: 10.1371/journal.pone.0221924 (PMC6776398; doi:10.1371/journal.pone.0221924)
Supplement: S2 Table — (DOCX) [file pone.0221924.s007.docx]

| **Table S2: Baseline characteristics of patients treated with rifampicin** | | |
| --- | --- | --- |
|  | **CD** | **Control** |
| Total number of patients | 19 | 8 |
| Mean age, yr (SD) | 44 (16.6) | 62 (3.5)* |
| Gender (M/F) | 5/14 | 3/4 |
| Mean duration of disease, yr (SD) | 13 (8.8) | - |
| # Smoking (%Yes) | 4 (25) | 2(33) |
| # Familiar IBD (%Yes) | 5 (36) | - |
| Concomitant medication: |  | |
| - none | 1 | - |
| - aminosalicylates | 3 | - |
| - corticosteroids | 4 | - |
| - immunosuppressives | 9 | - |
| - biological | 5 | - |
| # Biopsies colon | 106 | 36 |

* The healthy controls are significantly older than the CD patients p=0.002.
