# Supplementary material for: Pregnane X receptor activation constrains mucosal NF-κB activity in active inflammatory bowel disease
Source: PLoS One. 2019 Oct 3;14(10):e0221924. doi: 10.1371/journal.pone.0221924 (PMC6776398; doi:10.1371/journal.pone.0221924)
Supplement: S3 Table — (DOCX) [file pone.0221924.s008.docx]

| Table S3: Baseline characteristics of patients treated with linoleic acid | | | | |
| --- | --- | --- | --- | --- |
|  | Linoleic acid | | Linoleic acid +/- Rifampicin | |
|  | CD | Control | CD | Control |
| Total number of patients | 2 | 3 | 2 | 3 |
| Mean age, yr (SD) | 38(7.7) | 49(12.9) | 50(0.7) | 45(6.4) |
| Gender (M/F) | 1/1 | 2/1 | 1/1 | 2/1 |
| Mean duration of disease, yr (SD) | 17(7.1) | - | 5.5(0.7) | - |
| # Smoking (%Yes) | 0(0) | 0(0) | 0(0) | 1(33) |
| # with familiar link to IBD (%Yes) | 0(0) | - | 1(50) | - |
| Concomitant medication: |  | |  | |
| - none | 0 | - | 1 | - |
| - aminosalicylates | 1 | - | 1 | - |
| - corticosteroids | 0 | - | 0 | - |
| - immunosuppressives | 0 | - | 0 | - |
| - biological | 1 | - | 0 | - |
| # Biopsies colon | 10 | 8 | 10 | 8 |
