# Supplementary material for: Pregnane X receptor activation constrains mucosal NF-κB activity in active inflammatory bowel disease
Source: PLoS One. 2019 Oct 3;14(10):e0221924. doi: 10.1371/journal.pone.0221924 (PMC6776398; doi:10.1371/journal.pone.0221924)
Supplement: Attachment — Submitted filename: Response to Reviewers.docx [file pone.0221924.s010.docx]

Journal Requirements:

1. When submitting your revision, we need you to address these additional requirements.

We had revised our manuscript and supplementary files according to the journal requirements.

2. Please include captions for your Supporting Information files at the end of your manuscript, and update any in-text citations to match accordingly. Please see our Supporting Information guidelines for more information: <http://journals.plos.org/plosone/s/supporting-information>.

The captions for supporting information files were included at the end of our manuscript.

3. We note that you have included the phrase “data not shown” in your manuscript. Unfortunately, this does not meet our data sharing requirements. PLOS does not permit references to inaccessible data. We require that authors provide all relevant data within the paper, Supporting Information files, or in an acceptable, public repository. Please add a citation to support this phrase or upload the data that corresponds with these findings to a stable repository (such as Figshare or Dryad) and provide and URLs, DOIs, or accession numbers that may be used to access these data. Or, if the data are not a core part of the research being presented in your study, we ask that you remove the phrase that refers to these data.

Due to the PXR staining of the endoderm are not core part of our study, we removed the sentence refer to the data.

Additional Editor Comments:

This is an interesting and significant study. It would be more interesting to show the cell types in the biopsies samples that express NFkB signaling or NFkB-dependent genes.

The human protein atlas (<https://www.proteinatlas.org/>) show the protein expression of NF-κβ in colon tissue.

| **Cell types** | **NF-κβ protein expression** |
| --- | --- |
| Endothelial cells | Low |
| Glandular cells | Medium |

In the tissue of IBD patients, previous studies indicate NF-κβ is mostly found in mucosal macrophages and epithelial cells ^1, 2, 3^.

| Immunofluorescence detection of activated NF-κB in the inflamed mucosa. Activated NF-κB (Cy3 fluorescence) was identified in the lamina propria or in crypts near the basal membrane in inflamed mucosa. In normal mucosa, only nonspecific autofluorescence was detected. (A) Normal mucosa. The crypts show autofluorescence (orange). No red fluorescence (activated NF-κB, Cy3) can be detected. (B) Diverticulitis. Specific red fluorescence is detectable mainly in the lamina propria (arrowheads), corresponding to nuclei of cells containing activated NF-κB. (C) Crohn's disease mucosa without macroscopic inflammation (score 0). Only autofluorescence can be seen. The autofluorescence in the lamina propria seems to be greater compared with (A) control specimens perhaps because of the deposition of collagen. (D) Crohn's disease with a low degree of inflammation (+). Activated NF-κB can be detected (arrowheads). (E) Ulcerative colitis without inflammation (0). Again mainly autofluorescence is visible. (F–H) Ulcerative colitis in a severe (+++) state of inflammation. A large number of stained nuclei is detectable (arrowheads). Positive cells are located in the lamina propria and in the crypts (original magnification: A–F, 400×; G, 250×; and H, 1000×). | NF-κB activation in uninflamed and inflamed areas of the same patients. Specimens were prestained only with eosin. Activated NF-κB was stained with the α-p65mAb and BDHC (blue granular reaction product). In uninflamed mucosa, the number of activated cells was clearly lower. An obviously greater number of activated cells (arrows) is present in the inflamed areas. (A) Nonspecific colonic inflammation (inflammation grading, 0). (B) Nonspecific colonic inflammation (inflammation grading, +). Activated NF-κB is clearly present in the basal nuclei of crypt epithelial cells (basal membrane marked). (C) Crohn's disease (inflammation grading, 0). (D) Crohn's disease (inflammation grading, +++). Activated NF-κB is found close to small blood vessels (red-stained erythrocytes). (E) Ulcerative colitis (inflammation grading, 0). (F) Ulcerative colitis (inflammation grading, +++). Activated NF-κB is again visible close to small blood vessels. |
| --- | --- |

Joo Sung Kim *et al.* in their study mentioned the different characteristics of high and low NF-κβ activity patient groups (table below).

Han YM *et al.* (2017) NF-kappa B activation correlates with disease phenotype in Crohn's disease. PLoS ONE 12(7): e0182071.

When staining PXR in biopsies (not the same biopsies that we used for the stimulation experiments), the inflamed intestinal tissue show relatively low PXR expression (see below). Interestingly, the PXR expression is mainly found in epithelial cells.

Ctr is a biopsy from healthy individual; IA is inactive IBD; A is active IBD; ISC is ischemic colitis, IFC is infectious colitis; SB is small bowel

Review Comments to the Author

Reviewer #1: The study investigates the importance of PXR signaling in inhibiting NF-kB activity in the intestinal epithelial compartment during the IBD. Using colonic biopsies from control or IBD patients and several cell culture models, the authors observed a strict inverse correlation between colonic epithelial PXR levels and NF-kB target gene expression. The results are consistent with the well-established crosstalk between PXR and NF-kB signaling pathways. However, this study lacks mechanistic insight and did not provide further information on the known crosstalk. In addition, figure legends were misplaced, and grammar errors and typos were found throughout the manuscript.

Specific comments:
1. As mentioned in the method “Biopsies”, the biopsies were taken from the ascending colon, the transversum and the descending colon. Do these parts of colon show different expression patterns of PXR or activation pattern of NF-kB? Which part did the authors choose for the further study like “Stimulation of the biopsies”?

The different parts of colon show similar expression patterns of PXR and NF-κβ. Mostly the ascending colon and the transversum colon were used for further study and if they are not available, terminal ileum for small bowel would be taken instead. The detail information was shown in supplementary table 4.

2. To further investigate the crosstalk between PXR and NF-kB signaling, patients were stratified into three groups according to their PXR mRNA expression following Rifampicin treatment. The detailed information about PXR expression levels should be provided.

We show the detailed information of PXR expression level with Rifampicin treatment in supplementary file (Fig S3).

3. When the authors investigate the effects of NF-kB signaling on the PXR expression and activation, the expression levels of more PXR target genes should be measured.

Cyp3a4 and more genes were measured in supplementary figure 4 and supplementary figure 5.

And we also investigated the crosstalk between PXR and NF-kB signalling directly in the organoid derived from IBD patients (Fig 1G and 1H) (Fig 2G).

4. IL-1β protein levels were not shown in Figure 1 but was mentioned in line 190.

We measured protein level of IL-8 and mRNA level of IL-1β. Therefore we removed the phrase related with IL-1β protein levels.

5. Missing labels in Figure 2 panel F.

Labels of Figure 2F had been added.

6. Some typos were also found: e.g. “Rifamcipin” in line 131; “37 。C” in line 278.

Mistakes were corrected.

Reviewer #2: This is an interesting study, demonstrating that PXR levels are inversely correlated with NF-kappa B activity in the intestinal epithelial cells, conversely NF-kappa B signaling down-regulates PXP expression. Human tissues, intestinal organoids, and cell culture models were studied. The study suggests that PXR may have the potential to be a therapeutic target to inhibit NF-kappa B activity and gut inflammation. I have a few comments.

1. The abstract is not very informative. It would be helpful to expand the Results section of the Abstract. If word limit is a concern, shorten the Methods section.

Abstract was revised to expand the results section to reveal more information.

2. Fig. 2B: is p value > 0.01 or < 0.01? Fig. 2 F, the x-axis label is missing?

Figure 2B is p<0.01 and were corrected. Labels of Figure 2F had been added.

3. The description of Fig. 3 (Lines 285 to L295) appears not clear. Please reconcile the description with the figure.

We reorganized the description of Fig 3.

4. Please check mistakes through the paper, i.e. L69-L70; L202; L241.

Mistakes were corrected.

**Reference**

1.	Atreya I, Atreya R, Neurath MF. NF-kappaB in inflammatory bowel disease. *J Intern Med* 2008, **263**(6)**:** 591-596.

2.	Rogler G, Brand K, Vogl D, Page S, Hofmeister R, Andus T*, et al.* Nuclear factor kappaB is activated in macrophages and epithelial cells of inflamed intestinal mucosa. *Gastroenterology* 1998, **115**(2)**:** 357-369.

3.	Han YM, Koh J, Kim JW, Lee C, Koh SJ, Kim B*, et al.* NF-kappa B activation correlates with disease phenotype in Crohn's disease. *PLoS One* 2017, **12**(7)**:** e0182071.
